# Two waves of photosymbiosis acquisition in extant planktonic foraminifera explained by ecological incumbency

**DOI:** 10.1093/ismejo/wrae244

**Published:** 2024-12-11

**Authors:** Haruka Takagi, Yasuhide Nakamura, Christiane Schmidt, Michal Kucera, Hiroaki Saito, Kazuyoshi Moriya

**Affiliations:** Department of Marine Ecosystem Science, Atmosphere and Ocean Research Institute, The University of Tokyo, Chiba 277-8564, Japan; Department of Earth Sciences, Faculty of Science, Chiba University, Chiba 263-8522, Japan; Environmental Change Division, Estuary Research Center, Shimane University, Matsue 690-8504, Japan; Department of Botany, National Museum of Nature and Science, Tsukuba 305-0005, Japan; MARUM - Center for Marine Environmental Sciences, University of Bremen, Bremen 28359, Germany; Department Geoinformation, Helmholtz Centre Potsdam – GFZ German Research Centre for Geosciences, Potsdam 14473, Germany; MARUM - Center for Marine Environmental Sciences, University of Bremen, Bremen 28359, Germany; Center for International Research Collaboration, Atmosphere and Ocean Research Institute, The University of Tokyo, Chiba 277-8564, Japan; Department of Earth Sciences, Faculty of Education and Integrated Arts and Sciences, Waseda University, Tokyo 169-8050, Japan

**Keywords:** photosymbiosis, planktonic foraminifera, 18S rRNA gene amplicon sequencing, molecular phylogeny

## Abstract

Photosymbiosis, a mode of mixotrophy by algal endosymbiosis, provides key advantages to pelagic life in oligotrophic oceans. Despite its ecological importance, mechanisms underlying its emergence and association with the evolutionary success of photosymbiotic lineages remain unclear. We used planktonic foraminifera, a group of pelagic test-forming protists with an excellent fossil record, to reveal the history of symbiont acquisition among their three main extant clades. We used single-cell 18S rRNA gene amplicon sequencing to reveal symbiont identity and mapped the symbiosis on a phylogeny time-calibrated by fossil data. We show that the highly specific symbiotic interaction with dinoflagellates emerged in the wake of a major extinction of symbiont-bearing taxa at the end of the Eocene. In contrast, less specific and low-light-adapted symbioses with pelagophytes emerged 20 million years later, in multiple independent lineages in the Late Neogene, at a time when the vertical structure of pelagic ecosystems was transformed by global cooling. We infer that in foraminifera, photosymbiosis can evolve easily and that its establishment leads to diversification and ecological dominance to such an extent, that the proliferation of new symbioses is prevented by the incumbent lineages.

## Graphical Abstract

**Graphical Abstract:**
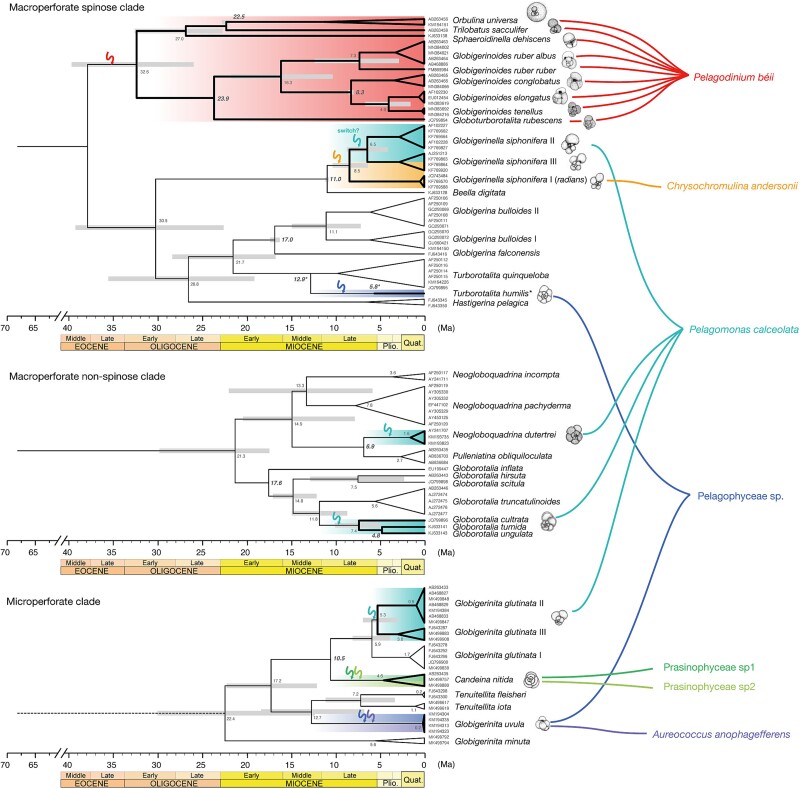

## Introduction

Photosymbiosis, a type of endosymbiosis involving photosynthetic microalgae, is an important source of evolutionary innovation that contributed to the radiation and ecological success of many lineages in broad spectra of eukaryotes [1–3]. Photosymbiosis is generally regarded as a mutualistic relationship that combines a heterotrophic host and photosynthesizing algae into a mixotrophic holobiont. This interaction entails symbionts providing photosynthetic products to the hosts, and in return, the host provides nutrients (metabolites) and shelter to the symbionts [4]. Although photosymbiosis occurs in several habitats, it has been widely observed as a successful adaptation in species inhabiting oligotrophic marine environments. In such environments, the mixotrophic holobiont can overcome nutrient constraints by internalized recycling and remineralization of particulate organic matter [3]. Notable examples of such holobionts are reef-building corals and other coral reef-associated organisms [5, 6]. In pelagic ecosystems, photosymbiosis is a common trophic strategy of planktonic protists, especially in warm, oligotrophic, sunlit surface oceans [7–11]. The large number of symbionts hosted in a single host cell effectively makes the holobiont a net primary producer, warranting a re-examination of the functional significance of the involved organisms [11].

Photosymbiosis is widespread among pelagic Rhizaria [9, 12]. Among planktonic foraminifera, responsible for the production of more than a quarter of pelagic biogenic carbonate deposited on the seafloor [13], photosynthesis carried out by the symbionts is also aiding their prolific biomineralization [14]. The occurrence of photosymbiosis in extant planktonic foraminifera was discovered using microscopy [15], 18S and 16S rRNA genes [16–19], and recently confirmed by the presence of photosynthesizing symbionts in 19 species [20]. A majority of them exhibited the persistence of symbiosis with the growth of the host. However, algal identity and diversity have not yet been elucidated, thus the specificity and evolutionary conservation of the symbiotic relationship and diversity of symbionts within a single host are unclear. This hinders us in gaining a better insight into the role of photosymbiosis in the evolution and ecological success of planktonic foraminifera.

Among photosymbiosis, there exist many examples where a single host is infected by different symbiotic taxa. For example, multiple diatom species were detected in benthic foraminifera [21–23]. In Acantharia, multiple haptophyte symbionts were identified [24], and an even wider combination of symbionts with dinoflagellates and haptophytes co-occurring in one host was also revealed [25]. Considering these studies, the analysis of symbiotic partnerships in planktonic foraminifera must consider flexibility and diversity. Therefore, in this study, we used single-cell 18S rRNA gene amplicon sequencing to investigate the diversity of planktonic foraminiferal symbionts by retrieving phototrophic components from the metabarcodes and analyzing their abundance and specificity patterns. We simultaneously analyzed photophysiological characteristics and responses to light environments of the photosymbiotic consortium to understand their ecological adaptability. By mapping the discovered patterns of specificity and identity of the symbionts on the phylogeny of the host clade, we distinguished conserved relationships from more recent symbiont acquisitions and determined the way specificity is linked to the age of the symbiotic relationship.

## Materials and methods

### Sampling

We obtained planktonic foraminifera specimens in the western North Pacific (Hakuho-Maru cruises KH-16-7 and KH-17-4 and TS Oshoro-maru cruise 243) and eastern North Atlantic (RV Meteor cruise M140) (Fig. S1, Table S1). We collected the samples by using vertical stratified towing (100-μm mesh for Hakuho-Maru and Meteor cruises and 63-μm mesh for Oshoro-maru cruise) or from the pumped seawater during the cruises (sampling depth, ca. 5 m, 100-μm mesh). We additionally collected some specimens from Sagami Bay by vertical towing using a 100-μm-mesh ring net to increase the taxonomic range of our analysis.

Specimens were identified to morphospecies level under a stereoscopic microscope. We included 19 morphospecies: 17 symbiotic species and 2 non-symbiotic species (Table 1). The specimens collected by the Hakuho-Maru and Meteor cruises were placed onto micropaleontological slides and preserved at −20°C until DNA extraction. Samples from the Oshoro-maru cruise were fixed using 99% ethanol onboard and preserved at 4°C until picking. The ethanol-fixed specimens were rinsed several times with milli-Q water before DNA extraction.

**Table 1:** Photosymbiotic partnerships revealed in this study.

Morphological species	Host species based on 18S rRNA gene amplicon sequences	Identified symbiont	*N*
*O. universa*	NA	*P. béii*	2
*T. sacculifer*	*T. sacculifer*	*P. béii*	10
*G. conglobatus*	*G. conglobatus*	*P. béii*	3
*G. tenellus*	*G. tenellus*	*P. béii*	2
*G. elongatus*	*G. elongatus*	*P. béii*	2
*G. ruber albus*	*G. ruber albus* (Ia)	*P. béii*	4
*G. ruber ruber*	*G. ruber ruber*	*P. béii*	4
*G. rubescens*	*G. rubescens*	*P. béii*	3
*G. calida*	*G. siphonifera* Ia (=*G. radians*)	*C. andersonii*	7
*G. siphonifera* Type I	*G. siphonifera* Ia (=*G. radians*)	*C. andersonii*	5
*G. siphonifera* Type II	*G. siphonifera* IIa3	*P. calceolata*	6
*T. humilis*	NA	Pelagophyceae sp.	7
*N. dutertrei*	*N. dutertrei*	*P. calceolata*	9
*G. cultrata*	*G. cultrata*	*P. calceolata*	8
*G. glutinata*	*G. glutinata* II/III	*P. calceolata*	17
*G. uvula*	*G. uvula* Ia	*A. anophagefferens*	4
*C. nitida*	*C. nitida* Ia	Prasinophyceae sp1/Prasinophyceae sp2	6
*G. bulloides*	*G. bulloides* NA/Ia	None	9
*N. pachyderma*	*N. pachyderma* II/VII	None	4

We divided *G. siphonifera* into two morphotypes (Type I and Type II) based on the criteria described previously [26, 27]. We differentiated *Globigerinella calida* from *G. siphonifera* based on the elongated chambers. Among the 112 analyzed single-cell foraminifera extractions, 96 yielded exploitable foraminiferal host sequences (Table S6). Host species based on 18S rRNA gene amplicon sequences are not always resolved into genotype level. We found no apparent inconsistency in morphologically identified species. However, a very small number of reads of different foraminiferal sequences were identified in some specimens (Table S1), which could have occurred due to the presence of contaminants or predation on the other foraminifera. Specimens morphologically assigned as *G. siphonifera* Type I and *G. calida* were genetically assigned to *G. siphonifera* Ia, which is suggested to be referred to as *G. radians* [28]. *N* shows the number of specimens analyzed for 18S rRNA gene amplicon sequencing.

### Fast repetition rate fluorometry

Prior to the fixation for DNA extraction, we used a number of specimens for photophysiological measurement by fast repetition rate (FRR) fluorometry. Some results were already reported [20], and the analytical method and details of the derived parameters can be found there. Briefly, among the photophysiological parameters of photosystem II (PSII) obtained by FRR fluorometry, we used the photosynthetic activity parameter *F_v_*/*F_m_* and functional absorption cross section σ_PSII_ as indicators of photosynthetic vitality and light absorption efficiency of symbionts, respectively.

The above photophysiological parameters were measured under dark conditions, which can be used to assess the potential/maximum performance of PSII chemistry. In contrast, measurement under a certain light condition enabled us to analyze the actual response of the PSII performance. Apart from the analyses above, four representative photosymbiotic species were used for the light response curve measurements. Selected specimens of *Trilobatus sacculifer*, *Globigerinella siphonifera* Type I, *Globorotalia cultrata*, and *Candeina nitida* collected from above 100 m onboard were measured by an FRR fluorometer to obtain a light curve with 12 sequential light intensity (*E*) from 10 to 1000 μmol m^−2^ s^−1^. Effective PSII quantum efficiency (*F_q_*’/*F_m_*’) was measured after exposure to each level of actinic light (450 ± 10 nm) for 5 min. Relative electron transport rate (*rETR*) was calculated as the product of *F_q_*’/*F_m_*’ and light intensity (*E* × *F_q_*’/*F_m_*’). We fitted the obtained *rETR*-light relationship to the photosynthesis-irradiance curve [29] to estimate the maximum electron transport rate (*rETR*_max_), the maximum light use efficiency (*α*), and the light saturation coefficient (*E_k_*) calculated as *rETR*_max_/*α*. The steeper slope (higher *α*) indicates a faster response to light increase, often resulting in lower saturation irradiance (*E_k_*). Therefore, higher *α* and lower *E_k_* generally indicate photoadaptation to low light, and vice versa.

### DNA extraction, amplification, and 18S rRNA gene amplicon sequencing

Isolated individuals were cleaned with a brush to ensure that any of the sticking environmental materials or possible commensal algae [30] were detached. Total DNA was extracted from foraminiferal individuals following the GITC* method [31]. We targeted the V9 region of the 18S rRNA gene, widely used in ecological studies for the investigation of eukaryotic diversity [32], with 1389F (5′-TTGTACACACCGCCC-3′)/1510R (5′-CCTTCYGCAGGTTCACCTAC-3′) primers. We applied the amplification method described previously [33]. Briefly, the reaction volume was 25.0 μl, containing 1.0 μl of extracted DNA, 12.5 μl of Q5 High-Fidelity 2× Master Mix (New England Biolabs), and 1.25 μl of 10 μM first fusion primers. An initial denaturation at 98°C for 1 min was followed by 35 cycles of denaturation at 98°C for 5 s, annealing at 65°C for 20 s and at 59°C for 10 s, extension at 67°C for 30 s, and a final extension at 67°C for 2 min. The first polymerase chain reaction (PCR) products were purified using AMPureXP (Beckman Coulter), and the DNA concentration was measured using Qubit 3.0 (Thermo Fisher). The second PCR amplification was performed with a 25 μl reaction mixture containing ca. 2.0 μl of the first PCR product (adjusted to contain 20 ng μl^−1^ of DNA), Q5 High-Fidelity 2× Master Mix, and fusion primers (including 8-mers indices and P5/P7 adapters) in the same final concentration as the first PCR. For the second PCR, an initial denaturation at 98°C for 30 s was followed by 15 cycles of denaturation at 98°C for 10 s, annealing at 72°C for 30 s, and a final extension at 72°C for 2 min. After the purification using AMPureXP, the length of the products was confirmed using TapeStation 4200 (Agilent Technologies). A run of sequencing was conducted using MiSeq (Illumina) with the MiSeq Reagent kit v3 (Illumina) following the recommended protocol.

We analyzed the data with Claident version 0.2.2016.07.05 [34] according to the manual. The forward and reverse sequences were first concatenated, and low-quality sequences (quality scores <30 on average) were removed. Chimera sequences were also excluded, and the rest of the sequences were clustered into operational taxonomic units (OTUs) (the minimum identification score was 0.97). We then taxonomically identified the OTUs using the TARA Oceans database (metabarcode database W4 [35]).

### Data analysis and statistics

From among the 112 single-cell extractions, a total of 3 892 911 sequence reads were retained after initial quality filtering. Each of the extractions yielded at least 14 675 reads. The retained reads could be clustered into 550 OTUs. After filtering out non-eukaryote, land plant, and land mammalian OTUs, 381 OTUs (3 076 162 reads) were retained, of which 45 OTUs (770 838 reads) were assigned to planktonic foraminifera and the remaining 336 OTUs (2 305 324 reads) to the other eukaryotes. In this study, we focused on phototrophic OTUs to investigate photosymbionts. Therefore, after removing the host OTUs, all other eukaryotic OTUs (heterotrophs that are possible prey or parasites) were only used to calculate the relative abundance of the phototrophs among all intracellular components. We assigned the trophism (phototroph/heterotroph) of the obtained OTUs based on the trophism category annotated for each reference barcode in the TARA database [35]. 62 OTUs (1 455 756 reads) were assigned as phototrophic, which were subsequently used for compositional analysis (Table S2). To understand the taxonomic position of the intracellular algae, we grouped the phototrophic OTUs based on their pairwise identity (ca. >95%), and reconstructed maximum likelihood phylogenetic trees with reference sequences similar to the obtained OTUs. We call these groups informative taxonomic groups (Fig. S2, Table S3).

We compared the OTUs identified as planktonic foraminifera to the sequences in the PFR2 database [36], and for microperforate and *Globigerinoides* species, we applied the most recent classification [37, 38].

All statistical analyses were performed using R 3.6.1 [39]. Alpha-diversity (Shannon index and OTU richness) of the phototrophs was calculated after rarefying the OTU data into the smallest number of sequence reads of phototrophs per specimen except those for *Globigerina bulloides* (336 reads). As *G. bulloides* is used as a representative non-symbiotic and carnivorous species, the sequence reads of phototrophs obtained from this species are extremely low (the smallest number of phototrophs is 1 out of 39 630 eukaryotic reads, FRM87), which makes comparison of phototroph diversity on an equal basis difficult. Beta-diversity [40] was calculated as the multivariate dispersion of samples within a foraminifera species. Here, phototroph communities in each individual were ordinated in multivariate space, and the dispersion of individuals within each foraminifera species was used as a metric of the flexibility of the phototroph composition. Specifically, principal coordinate analysis of Bray–Curtis distances of square-root-transformed compositional data was used to calculate distance-to-centroid values using the betadisper function in the vegan package [41]. Rarefaction curve analysis was performed on phototrophic reads to examine the saturation level of phototrophic OTUs for each species.

A nonmetric multidimensional scaling (NMDS) plot was produced using the Bray–Curtis distance matrix to visualize the compositional difference of the phototrophic metabarcodes among the individual foraminifera. To test the proportional differences among foraminifera species, permutational analysis of variance (PERMANOVA) was conducted using the adonis function in the vegan package. To compare the differences in the diversity indices (Shannon index, rarefied richness, and beta-diversity) among species and photophysiological parameters (*F_v_*/*F_m_*, σ_PSII_, *rETR*_max_, *α*, and *E_k_*) among the algal groups, statistical tests for comparison of differences in medians (Kruskal–Wallis test and post hoc Nemenyi test for multiple comparisons) were conducted.

### Phylogeny and molecular clock analysis of planktonic foraminifera

Previous molecular phylogenetic studies for spinose planktonic foraminifera have suggested several possible placements of dinoflagellate-bearing species [42–44]. Specifically, de Vargas et al. [42] showed the inconsistent position of the clade *Globigerinoides ruber* + *Globigerinoides conglobatus* that is related either to the clade of *Globigerinella* in their ML tree, or to clade *T. sacculifer* + *Orbulina universa* in their NJ tree. In contrast, Aurahs *et al.* [43] showed consistent monophyly of *G. ruber*, *G. conglobatus*, *T. sacculifer*, and *O. universa* in their phylogeny reconstructed based on several alignment algorithms. Most recently, in the more comprehensive study of Morard *et al.* [44], *Globigerinoides* (*Globigerinoides* + *Globoturborotalita rubescens*) formed a sister clade with *Globigerinella* + *Beella* clade, and *T. sacculifer* + *O. universa* clade formed a sister clade with *Turborotalita*. In this study, we tested four possible topologies of 16 spinose planktonic foraminifera, focusing on the position of dinoflagellate-bearing taxa: a monophyly, two patterns of paraphyly, and a polyphyly (Fig. S3). We compared the topologies based on the bootstrap proportion using the RELL method [45], the weighted Shimodaira–Hasegawa test [46], expected likelihood weights [47], and the approximately unbiased test [48] implemented in IQ-TREE v.1.6.12 [49].

To reconstruct the evolutionary history of planktonic foraminifera, we applied a molecular clock estimation using a subset of curated 18S rRNA gene partial sequences in the public database (105 representative sequences from 33 morphospecies, PFR2 [36]). Considering the possibility of their different origins from benthic ancestors, we analyzed the three clades of planktonic foraminifera—macroperforate spinose group (51 sequences, 1352 sites), macroperforate non-spinose group (26 sequences, 1402 sites), and microperforate group (28 sequences, 1077 sites)—separately. The sequences were aligned using MEGA7.0 [50] and corrected manually. Bayesian phylogenetic analysis was performed using the Yule’s model within the BEAST2 v.2.6.7 framework. We used the divergence between *Orbulina* and *Trilobatus* (22.0–23.0 Ma [51]), between the genus *Beella* and *Globigerinella* (10.8 Ma [28]), between *Globoturborotalita rubescens* and genus *Globigerinoides* (23.7 Ma [38, 52]), and used the first appearance datum (FAD) of *G. conglobatus* (8.0–8.6 Ma [53]), *Neogloboquadrina dutertrei* (6.9 Ma [52]), *Globorotalia inflata* lineage (17.5 Ma [54]), *Globorotalia tumida* and *Globorotalia ungulata* (4.3–5.8 Ma [54]), and *C. nitida* (10.5 Ma [53]), which are known from the fossil record to constrain the ages. We specified the relaxed-clock lognormal model, which assumes that the substitution rates for branches are independent variables from a lognormal distribution, and the General-Time Reversible model as the substitution model. Markov-chain Monte Carlo (MCMC) analyses were conducted for 200 million generations, and removed 10% of the chain as burn-in. The tree was saved at every 10 000th generation. The convergence of the MCMC runs was assessed by using Tracer v1.7.2. The tree sample was summarized using TreeAnnotator v2.6.7 as a maximum-clade-credibility tree, with 10% of samples discarded as burn-in and median heights used as the node heights in the tree.

## Results

### Phototrophic OTUs and their abundance, composition, and diversity

Rarefaction curves showed that the number of phototrophic OTUs in the non-symbiotic species (*G. bulloides* and *Neogloboquadrina pachyderma*) increased rapidly without saturation, contrasting with the gradual saturation of only seven OTUs at maximum in the symbiotic species (Fig. S4). Being unsaturated illuminated the high diversity of intracellular phototrophs in non-symbiotic species.

**Figure 1:**
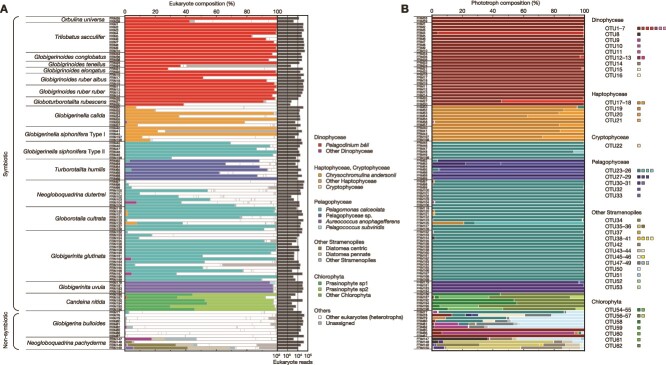
Taxonomic compositions of eukaryotic reads focusing on the phototrophic components. (A) The informative taxonomic group level (see text for detail). The total numbers of eukaryotic reads are also represented in the right column. Host reads are excluded. Heterotrophic reads (non-phototrophic components) are not discussed further in this study. (B) OTU-based phototrophic reads. Each color corresponds to a single OTU. Detailed information on OTUs is provided in Table S2.

**Figure 2:**
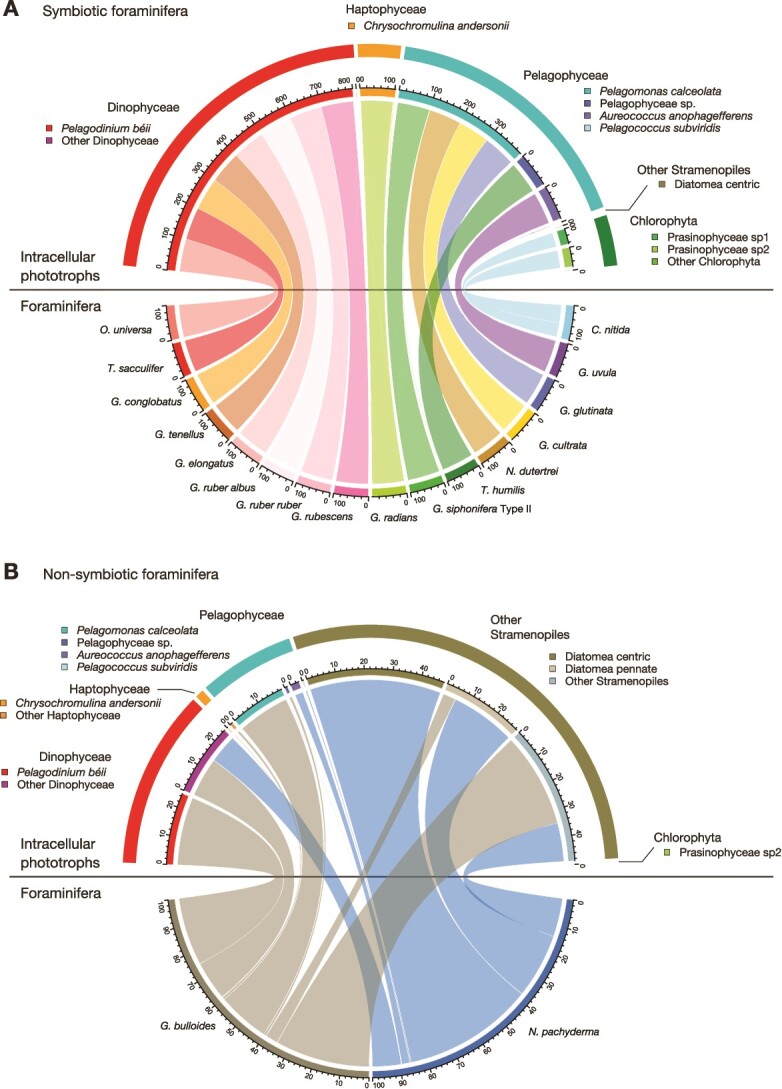
Chord diagram showing the bipartite relationship between foraminifera and phototrophs. (A) Symbiotic foraminifera. (B) Non-symbiotic foraminifera. In this visualization, the dataset for each foraminiferal species was converted to proportions, and the phototrophic OTUs were grouped into the informative taxonomic groups.

**Figure 3:**
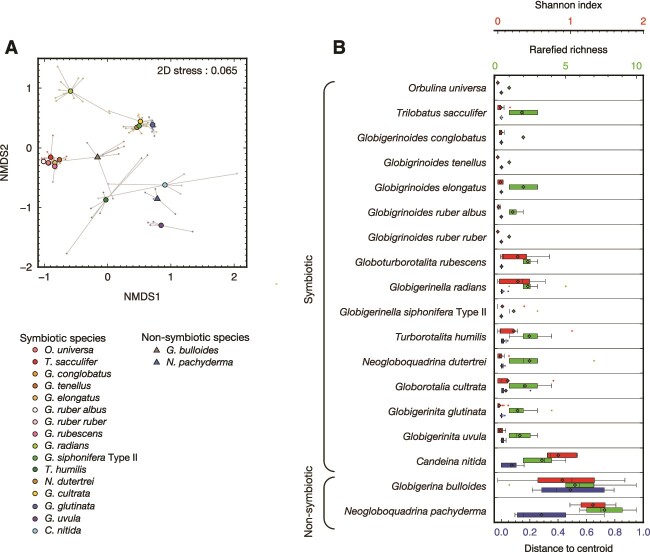
Compositional differences of intracellular phototrophs among foraminifera. (A) NMDS ordination plot. Bray–Curtis distances between samples based on OTU composition grouped by foraminifera species are used. Small points are samples (foraminifera individuals) and large circles are the centroid for each species. (B) Diversity indices for each species. Alpha-diversity (Shannon index and rarefied richness) and beta-diversity (distance-to-centroid in the principal coordinate ordination plot of Bray–Curtis distances) of phototrophic compositions for each species are shown. Box plots represent first and third quartiles as hinges, and the midlines represent medians. Means are also represented with diamonds. Values more than 1.5 times the length of the box from either end of the box are considered outliers.

The dominance of the following seven informative taxonomic groups was observed in certain foraminiferal groups: *Pelagodinium béii* (Dinophyceae), *Chrysochromulina andersonii* (Haptophyceae), *Pelagomonas calceolata*, Pelagophyceae sp., *Aureococcus anophagefferens* (Pelagophyceae), Prasinophyceae sp1, and Prasinophyceae sp2 (Chlorophyta) (Figs 1 and 2). We represented the overall compositional dissimilarities in an NMDS ordination, which presents clusters by the primary symbiont they possess (Fig. 3A). PERMANOVA showed no significant difference in phototroph composition among the species bearing *P. béii* (*P* = .17) and among the species bearing *P. calceolata* (*P* = .46).

Alpha-diversity of intracellular phototrophs was higher overall in non-symbiotic species than in symbiotic species (Fig. 3B). Beta-diversity, which represents multivariate dispersion of phototroph composition from the replicate specimens belonging to the same host species, was also higher in non-symbiotic species than in symbiotic species (Fig. 3B). Among the symbiotic species, the Shannon index of *C. nitida* was significantly higher than that of the three symbiont-bearing species, *G. ruber ruber*, *O. universa*, and *G. glutinata* (*P* < .05, Table S4), but in contrast, the beta-diversity of this species was low, with no significant difference between the other symbiotic species.

### Photophysiological states

**Figure 4:**
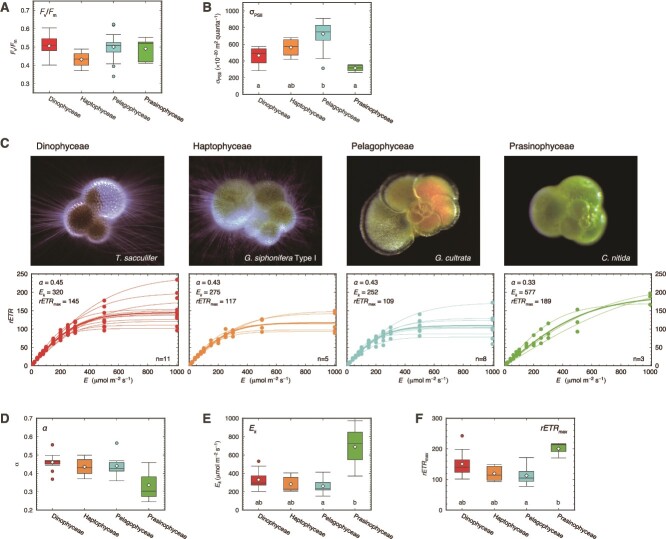
Photophysiological features summarized by symbiotic algal types. (A) *F_v_*/*F_m_*. (B) σ_PSII_. (C) Light response curves with *E* of 10, 20, 40, 60, 80, 100, 150, 200, 250, 300, 500, and 1000 μmol m^−2^ s^−1^. Dinophyceae (results of *T. sacculifer*), Haptophyceae (results of *G. siphonifera* Type I), Pelagophyceae (results of *G. cultrata*), and Prasinophyceae (results of *C. nitida*). Dots and thin lines represent individually measured values and fitted curves, and bold lines are representative curves for species fitted using mean values for all light intensities. The values of parameters shown are for the representative curves. A representative image of each species is shown above each panel (not scaled). Summary of the three light response curve parameters; (D) *α*, (E) *E_k_*, and (F) *rETR*_max_. Box plots represent first and third quartiles as hinges, and the midlines represent medians. Means are also represented with diamonds. Values more than 1.5 times the length of the box from either end of the box are considered as outliers. Statistically significant differences are indicated by letters (Kruskal–Wallis test and post hoc Nemenyi test, *P* < .01).

We grouped the obtained photophysiological parameters by symbiont taxonomy (Dinophyceae, Haptophyceae, Pelagophyceae, and Prasinophyceae). There was no significant difference between an indicator of photosynthetic activity, *F_v_*/*F_m_*, of Dinophyceae, Haptophyceae, Pelagophyceae, and Prasinophyceae (Kruskal–Wallis test, *P* = .16; Fig. 4A). Conversely, σ_PSII_, an indicator of light absorption efficiency, was significantly different (Kruskal–Wallis test, *P* < .01; Fig. 4B). The post hoc pairwise comparisons identified the highest σ_PSII_ value in Pelagophyceae and the lowest in Prasinophyceae. We also analyzed the light response curves of selected species—*T. sacculifer*, *G. siphonifera* Type I, *G. cultrata*, and *C. nitida*—representing each of the four algal types of photosymbiosis. The light curve measurements showed high intra-species variability (Fig. 4C). However, a slower light response was evident in Prasinophyceae-bearing species *C. nitida*. When we compared the slope parameters, the initial slope indicating the maximum light use efficiency (*α*) was lower in Prasinophyceae than that in the others, although the difference was not statistically significant (Kruskal–Wallis test, *P* = .22, Fig. 4D). Both the light saturation coefficient (*E_k_*) and the maximum electron transport rate (*rETR*_max_) were statistically different among the four groups (Kruskal–Wallis test, *P* = .04 for *E_k_* and *P* = .02 for *rETR*_max_, Fig. 4E–F). A pairwise comparison found a significant difference in *E_k_* between Prasinophyceae and Pelagophyceae (Nemenyi test, *P* = .04, Fig. 4E), indicating the high-light-adapted nature of Prasinophyceae and contrasting low-light-adapted nature in Pelagophyceae. Likewise, the difference in *rETR*_max_ was also significantly high in Prasinophyceae and low in Pelagophyceae (Nemenyi test, *P* = .03, Fig. 4F).

### Phylogenetic inferences

**Figure 5:**
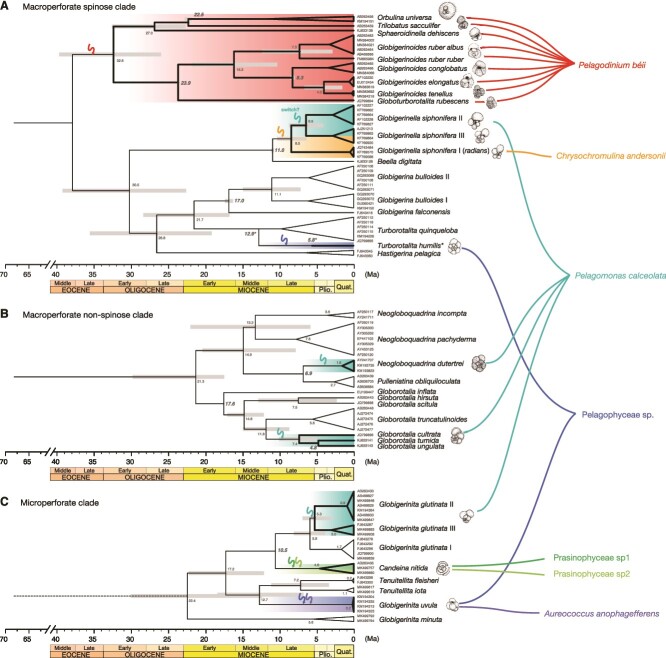
Molecular phylogeny and estimated divergence time of modern planktonic foraminifera with identified symbiont identity. (A) Macroperforate spinose clade. (B) Macroperforate non-spinose clade. (C) Microperforate clade. We manually added *T. humilis* due to the lack of available sequence with enough length following the topology used in previous studies: the divergence between *T. quinqueloba* and *T. humilis* (12.9 Ma [44]) and FAD of *T. humilis* (6.0 Ma [52]). We italicized ages used as calibrations in bold character. Symbiotic clades have been presented using bold lines, hatched by colors coded by symbiont species. The timings of possible symbiosis acquisition are indicated by arrows on the branch next to the node of the symbiotic clade.

Our comparison of the four tested topologies identified the monophyly of the dinoflagellate-bearing clade as the most likely scenario (Fig. S3). The clocked phylogeny also inferred a monophyletic topology for the dinoflagellate-bearing clade (Fig. 5A). The reconstructed phylogeny of the remaining clades implied the occurrence of further events of photosymbiosis acquisition in all three main clades of planktonic foraminifera (Fig. 5). In each clade, photosymbiosis was acquired several times. Assuming that photosymbiosis was acquired at some point on the common ancestral branch of a single photosymbiotic clade, it can be inferred that the evolution of modern photosymbiosis occurred independently at least eight times. The earliest acquisition event dates back to the Oligocene (32.6 Ma in divergence time) in the dinoflagellate-bearing clade; however, for the other symbiotic clades, the divergence times are earlier than 8.5 Ma and do not date back to the middle Miocene, even when considering the large credible interval on the divergence time of neighboring branches. The mid-point ages of divergence of all other symbiont-bearing branches (arrows in Fig. 5) are dated to 4–10 Ma, i.e. from the late Miocene to the early Pliocene.

## Discussion

Intracellular DNA extracted from foraminiferal cells and identified as belonging to phototrophs can either represent photosymbionts or incompletely digested prey. In the known non-symbiotic foraminifera (*G. bulloides* and *N. pachyderma*), all detected phototrophs must represent prey. This is supported by the fact that the specimens of *G. bulloides* that were used for DNA analysis showed no active chlorophyll fluorescence (Table S1), indicating the loss of photosynthetic capability of the detected phototrophs during digestion. In fact, the previous study reported the presence of living cyanobacteria within *G. bulloides* (genetic type IId) [18], but these observations have not yet been confirmed by measuring active photosynthesis. In our study, although we targeted eukaryotic algae using the FRR measurements with blue excitation wavelength (450 nm), which is not optimal for analyzing cyanobacteria (>500 nm), this method can also detect active chlorophyll fluorescence of cyanobacteria if any. Therefore, the non-detection of active chlorophyll fluorescence confirmed that *G. bulloides* used in our study did not possess any photosynthesizing cyanobacteria as well. Overall, the notable feature of phototroph composition in non-symbiotic species was the high diversity within one individual (alpha-diversity) and high variability among specimens of the same species, reflecting the composition of the algal community on which they had fed (beta-diversity) (Fig. 3B). In contrast, phototrophs found in symbiotic species showed close and consistent relationships with their host with low alpha- and beta-diversity (Figs 2 and 3B). In those species, seven phototroph taxa account for >50% of total eukaryotic reads: *P. béii*, *C. andersonii*, *P. calceolata*, Pelagophyceae sp., *A. anophagefferens*, Prasinophyceae sp1, and Prasinophyceae sp2 (Fig. 1). Such high abundance and specificity, along with the fact that some species had already been reported as symbionts of planktonic foraminifera were sufficiently persuasive evidence that these taxa represent the symbionts. Although the other phototrophic taxa in the symbiotic foraminifera could represent prey, we cannot reject the possibility that they indicate the existence of a more diverse symbiont consortium. Some specimens seem consistently associated with two phototrophic taxa that act as photosymbionts (Table S2). *Globigerinella radians* possessed few *P. calceolata* together with its primary symbiont *C. andersonii*. *Turborotalita humilis* also possessed *P. calceolata* in addition to its primary symbiont Pelagophyceae sp., and some specimens of *G. cultrata* possessed *C. andersonii* together with *P. calceolata*. If the lesser number of symbiotic taxa could also serve as symbionts, it is possible that planktonic foraminifera can also engage in flexible photosymbiosis like their benthic relatives [21, 23].

Our study confirmed several interesting co-ownerships of the symbionts between foraminifera and radiolarians—*P. béii* was recently discovered from Acantharia [25], *C. andersonii* was recently described as a prymnesiophyte symbiont from a polycystine radiolarian *Dictyocoryne truncatum* [55], and Prasinophyceae sp1 forms a monophyletic group together with a symbiont previously reported from a radiolarian *Spongaster tetra* [56] (Fig. S2)—indicating that the genetic pool of algal symbionts is shared within pelagic Rhizaria. Previous studies have suggested that symbioses in pelagic realms differ from those seen in coastal environments such as coral reefs in terms of the population size of free-living and *in hospite* symbionts [57]. In pelagic oceans, the majority of the symbiont population is in the free-living stage, and only a small portion of symbionts is in a symbiotic state, “exploited” by the hosts [57]. TARA Ocean data [35] of the seven symbionts determined in this study confirmed their large population sizes in free-living stage; therefore, establishing symbiosis with those taxa can be said to be stochastically easy. A theoretical study previously suggested that—when the free-living population is large enough—mutualism is easily maintained without vertical transmission [58]. To our best knowledge, foraminiferal hosts digest their symbionts at the onset of gametogenesis [59, 60]; thus, the symbionts cannot be transferred vertically unless the host undergoes asexual reproduction [61]. The majority of reproduction in planktonic foraminifera is sexual; through gametes release followed by their fusion. No symbionts have been observed in gamete [62–64]; therefore, the generational reacquisition is mandatory. *P. béii* is a common alga in the ocean (Fig. S5), and the dinoflagellate-bearing symbiosis is maintained in a single clade for a long time (Fig. 5A); these two facts suggest that this is an example of a successful mutualism without vertical transmission.

In this study, we highlight the difference in photophysiological features, especially between the newly discovered prasinophyte symbionts (high-light adapted) and pelagophyte symbionts (low-light adapted). Even when the collection depth, thus the light intensity difference of their collected environment, is taken into consideration, the difference among the light adaptation of the symbionts is clear (Table S5). In fact, the contrasts in light response characteristics between green and brown algal symbionts are similar in planktonic and benthic foraminifera. In symbiotic larger benthic foraminifera, chlorophyte-bearing soritids showed a higher light preference than those of diatom-bearing amphisteginids [65] and showed narrower and shallower depth distribution [66]. The light adaptability revealed in this study also appears to mirror the general habitat depth for each planktonic host. The abundance peak of *P. calceolata*-bearing species *N. dutertrei* and *G. cultrata* often corresponds to the deep-chlorophyll maximum [67]. Considering that photosymbiotic planktonic foraminifera are mixotrophic, their living depth may be determined by the trade-off between phototrophy and heterotrophy. Generally, it can be assumed that the shallower the waters they inhabit, the more phototrophy-dependent they may be. However, once a species could evolve to establish a partnership with symbionts showing low light requirements like *P. calceolata* (Figs 4, S5, and S6), it could break this framework, widening its ecological adaptability in terms of nutrition.

Our results highlighted a high specificity of host–symbiont partnerships at the 18S rRNA gene V9 level resolution. Such specificity contrasts with the reported mode of symbiosis in other rhizarian hosts, involving multiple diatom taxa in larger benthic foraminifera [21, 23], multiple haptophyte symbionts in Acantharia [24], and an even greater variety of symbionts from distant taxonomic groups found in other Acantharia [9]. The notable ecological difference between planktonic and benthic foraminifera is that symbiotic benthic species reside in coastal regions where the environmental fluctuation is higher than that in pelagic open ocean environments. In such highly fluctuating environments, strict specificity of the algal partner can be a risk to the host’s survival; the chance they fail to meet the right symbiont may be high. Therefore, flexibility might be a reasonable strategy for adapting to frequent local environmental changes as seen in hermatypic corals that host several types of *Symbiodinium* clade in one host [6, 68]. The difference between symbiotic Acantharia and planktonic foraminifera, both mainly living in oligotrophic pelagic environments, must have other causes. We speculate that it is related to the number of symbiont recruitment events during the host’s lifetime, which vary between foraminifera and Acantharia. Multiple algal partners in Acantharia could be the result of multiple recruitments of different symbionts, whereas a single symbiont in planktonic foraminifera could reflect a single recruitment. This difference could be due to the symbionts’ ability to proliferate within the host. In planktonic foraminifera, mitotic cells have frequently been observed in their cytoplasm [69, 70], and the increasing number of symbionts and Chl *a* content with increasing host size has been well documented as further evidence of their ability to reproduce *in hospite* [20, 69]. However, in the case of haptophyte-bearing Acantharia, it has recently been shown that the symbiont cell division is blocked when they are in a symbiotic state [71]. The difference in the average number of symbiont cells per host—14.7 in Acantharia [72] compared to 10^3^–10^4^ cells in planktonic foraminifera [69]—may also reflect the different modes of symbiotic interaction. Specifically, symbionts in planktonic foraminifera can proliferate within the host, whereas in Acantharia, such proliferation does not seem to occur.

From the symbiont side, the same algal species are associated with multiple hosts, representing the low host specificity. *P. béii*, known as a foraminiferal symbiont [17, 64, 73], was detected in all species belonging to *Globigerinoides*, *Globoturborotalita*, *Trilobatus*, and *Orbulina*. The taxa bearing this exclusively hosted symbiont appear to show lower symbiont diversity, implying a highly specialized symbiosis. This specialization should also be related to the known metabolic interaction on carbon and nitrogen [70, 74] and also the diurnal rhythm of the symbiont distribution—outside of the test during day and inside during night [75]—that can optimize the symbiont photosynthesis and translocation of the organic substance. The taxonomic range of *P. calceolata*-bearing foraminifera is large, encompassing all three phylogenetic groups that evolved separately from different benthic foraminiferal ancestors (Fig. 5). As no benthic species are reported to host pelagophytes, it is plausible that the symbiosis with *P. calceolata* has developed separately, at least four times, in the history of planktonic foraminifera (Fig. 5). *Pelagomonas calceolata* is a ubiquitous constituent of marine picophytoplankton—from subtropical to subarctic [76]. The TARA Ocean data evidences the common occurrence of this species from various oceanic regions (Fig. S5). The ability of *P. calceolata* to inhabit various oceanic environments might be one of the factors that enables it to act as a symbiont. For a host that relies on horizontal symbiont transmission, ubiquitous species, such as *P. calceolata*, represent an ideal candidate symbiont. If the hosts of *P. calceolata* utilize it as a symbiont mainly because it is easy to access, it may have retained the capacity to establish symbiosis with other algae as well. The involvement of *P. calceolata* in all cases of potential secondary symbionts indicates that the partnership with this species is less specific than that between the globigerinoidids and *P. béii*.

On the time tree produced by the molecular clock analysis, we mapped the symbiotic relationships of species (Fig. 5). In the dinoflagellate-bearing clade, symbiont acquisition dates back at least to the early Oligocene. This is the oldest extant symbiotic clade, and the only one that includes multiple host genera, representing the most evolutionarily successful symbiotic lineage. Access to the rich fossil record of extinct clades of planktonic foraminifera allows us to evaluate the evolutionary significance of the emergence of this successful symbiosis. At the Eocene/Oligocene boundary, due to the global cooling associated with the development of a permanent ice-sheet on Antarctica, planktonic foraminifera experienced an immense reduction in the diversity of mixed-layer genera, including typical Eocene symbiotic genera *Acarinina*, *Globigerinatheka*, and *Orbulinoides* [52, 77]. Consistent with the concept of incumbency or evolutionary priority effect [78, 79], it may have been the demise of the Eocene surface-layer fauna that opened the surface ocean niche to new lineages, with new photosymbioses. The Oligocene ancestor of the modern dinoflagellate-bearing species, probably a group of *Globoturborotalita*, may have colonized the newly opened niche. The ancestral *Globoturborotalita* in the Eocene, *G. martini* was also presumed to be symbiotic [52, 80], hence the symbiosis with dinoflagellates could originally date back to the Eocene. At the time of *G. martini* appearance in the mid-Eocene, various other symbiotic lineages, such as *Acarinina* and *Globigerinatheka*, were still thriving in the surface waters. Diversification of symbiotic *Globoturborotalita* might have been prevented until the demise of the dominant photosymbiotic incumbent clades in the Oligocene (Fig. S7).

All extant non-dinoflagellate symbioses emerged some 20 million years later, in the late Neogene (Fig. 5). The multiple independent establishments of photosymbiosis in extant and extinct foraminifera indicate that becoming mixotrophic is evolutionarily easy for these protists, and the 20 million years gap in symbiosis acquisition after the Oligocene event may represent an establishment of a new incumbency.

In this model, the late Neogene proliferation of new symbioses would require a rearrangement of the pelagic habitat, resulting in the emergence of new niches suitable for algal symbiosis but not accessible to the dinoflagellate-bearing clade. Indeed, there is evidence that the gradual global cooling since the Miocene has promoted the expansion of planktonic foraminifera (and other plankton) into deeper habitats [81]. This has been attributed to the reduced rate of remineralization of organic matter due to the ongoing cooling, which improved the efficiency of organic matter transport to deeper layers in the surface ocean. It is interesting that among the four lineages where the new low-light adapted symbioses emerged is the genus *Globorotalia*, which is the most recently diversified genus of planktonic foraminifera [52]. In the course of this recent diversification, some species of *Globorotalia* have expanded their niche to the aphotic zone, but our results indicate that other species, such as *G. cultrata*, adopted a mixotrophic strategy with a pelagophyte, staying in relatively deeper parts of the photic zone and benefiting from phototrophs as well, thereby separating its niche from their deeper-dwelling non-symbiotic relatives, such as *Globorotalia truncatulinoides* and *Globorotalia hirsuta*.

Considering the following three characteristic points—(i) relatively lower specificity and less fixed partnership in the pelagophyte-bearing species, (ii) ubiquitous and large population size of free-living pelagophytes, and (iii) the recent repeated exploitation of pelagophytes by various foraminiferal hosts—we can state that pelagophyte symbionts are easy to obtain but not strictly fixed in the host lineage. In contrast, the long-lasting relationship between dinoflagellates is highly tuned, conservative, and persistent. If such specificity is linked to the age of symbiotic relationships, we have yet to recognize that symbiosis may have evolved and declined among various extinct lineages over a shorter period of time than we would expect. Early symbiosis establishment with dinoflagellates may also have been less fixed, eventually progressing toward more specificity. Either way, our results are consistent with an evolutionary scenario in which the emergence of mixotrophy leads to diversification and ecological success to an extent that the incumbent prevents the proliferation of new symbioses, unless new niche space becomes available. This implies that the loss of symbiotic lineages in planktonic foraminifera during extinction events can be rapidly compensated by the emergence of new symbioses, indicating functional resilience independent of taxonomic diversity.

## Supplementary Material

Supplementary_Figures_wrae244

Supplementary_Tables_wrae244

## Data Availability

The raw sequence reads are archived in the DDBJ Sequence Read Archive under accession number PRJDB18714. All the other data are available in the main text or the supplementary materials.
