# Supplementary material for: Two waves of photosymbiosis acquisition in extant planktonic foraminifera explained by ecological incumbency
Source: ISME J. 2024 Dec 11;19(1):wrae244. doi: 10.1093/ismejo/wrae244 (PMC11736160; doi:10.1093/ismejo/wrae244)
Supplement: Supplementary_Figures_wrae244 [file supplementary_figures_wrae244.pdf]

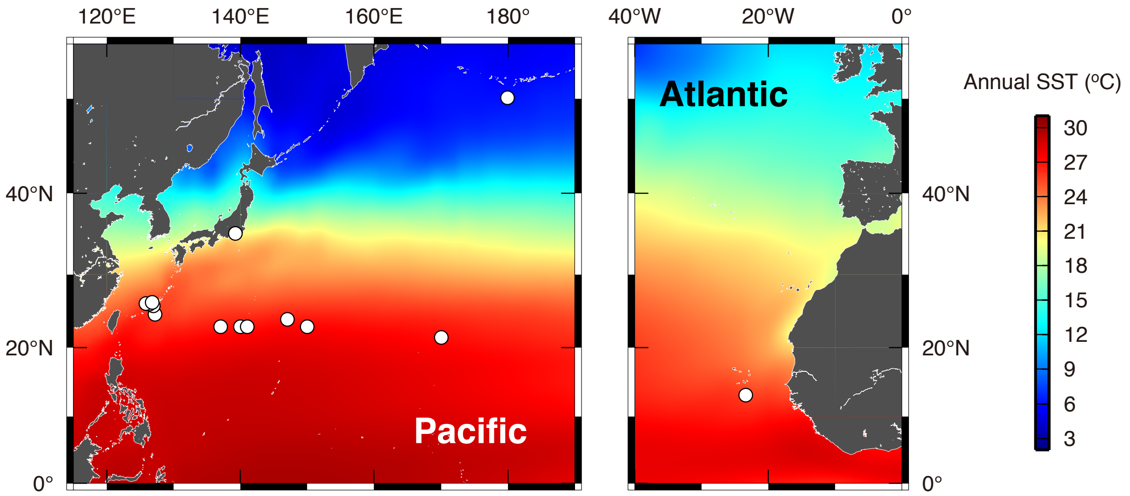

Fig. S1. Maps showing the sampling points. Annual sea surface temperature data were from World Ocean Atlas 2013 (Locarnini et al., 2013). See Table S1 for detailed sampling information.

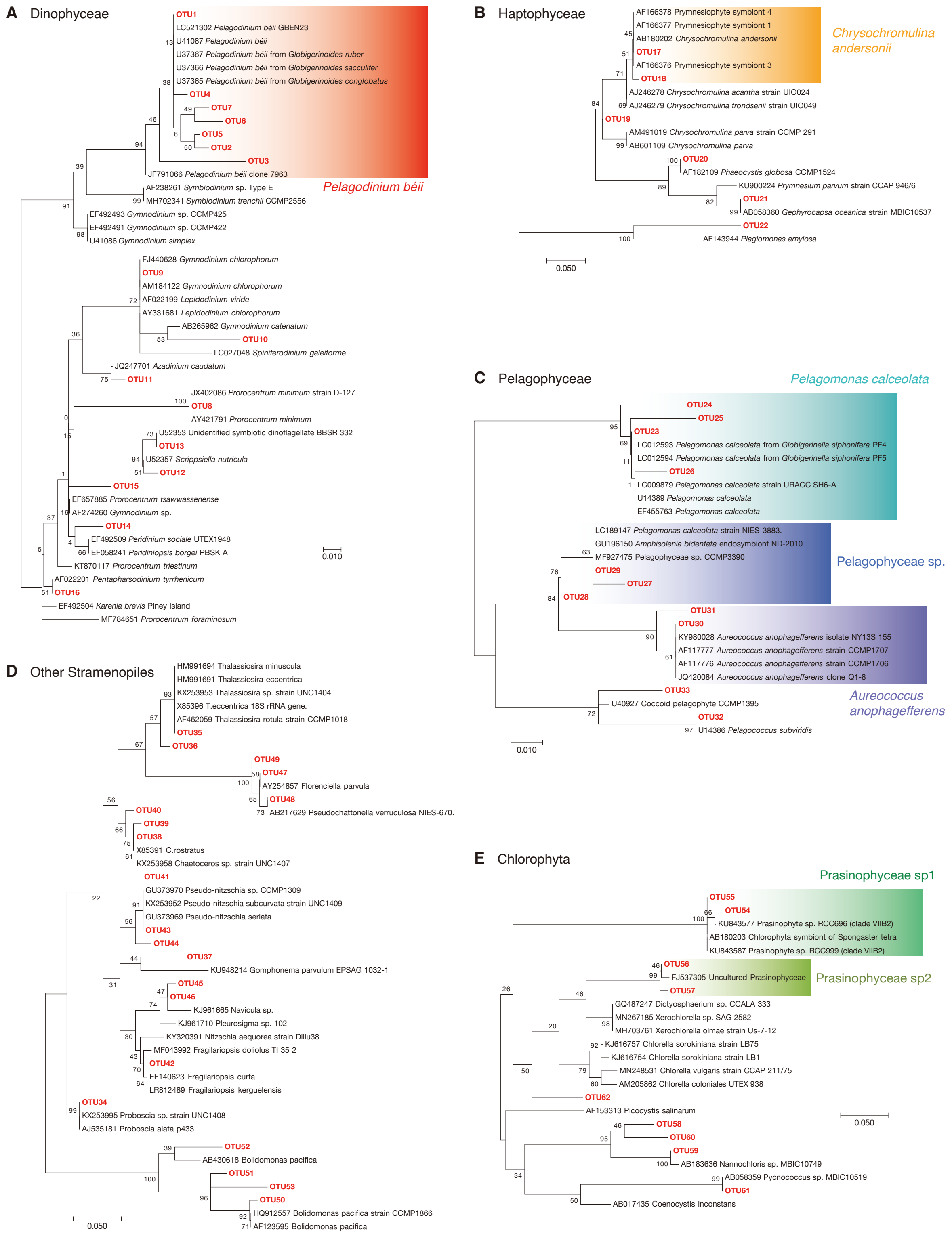

Fig. S2. Maximum likelihood trees based on 169 bp sequences showing the phylogenetic grouping of OTUs for each algal group. A, Dinophyceae, **B**, Haptophyceae, **C,** Pelagophyceae, **D,** Other Stramenopiles, and **E**, Chlorophyta. Box colors correspond to the ones used in Fig. 1.

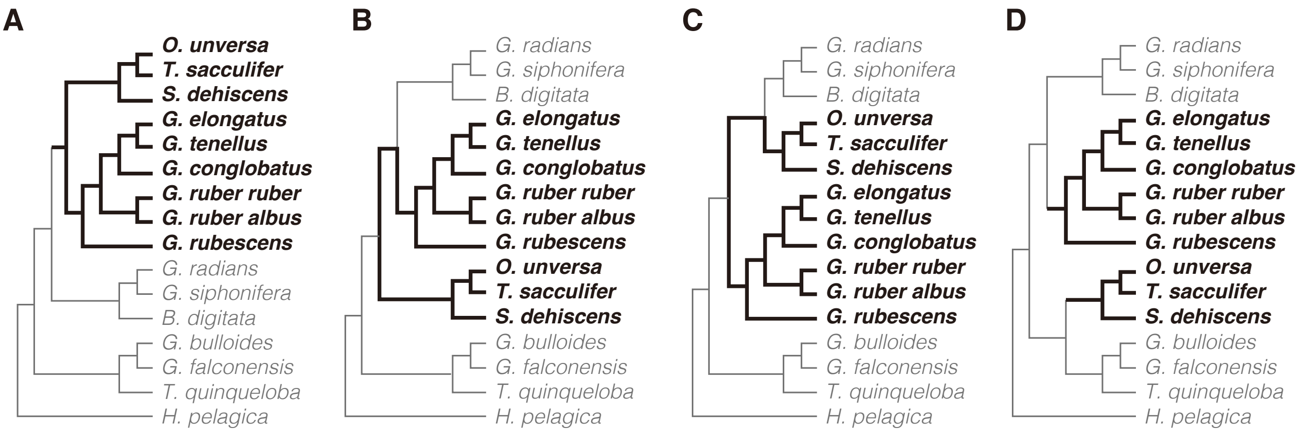

E

Tree	Topology	Δln L	bp-RELL	ELW	*p* (wSH)	*p* (AU)
a	((((((uni,sac),dehi),((((elong,tene),cong),(rubr,ruba)),rubes)),((radi,siph),digi)),((bullo,falco),quin)),pela);	0	0.969	0.953	0.999	0.977
b	(((((uni,sac),dehi),(((((elong,tene),cong),(rubr,ruba)),rubes),((radi,siph),digi))),((bullo,falco),quin)),pela);	9.718	0.0229	0.0287	0.0908	0.0354
c	(((((((elong,tene),cong),(rubr,ruba)),rubes),(((uni,sac),dehi),((radi,siph),digi))),((bullo,falco),quin)),pela);	9.841	0.0083	0.0181	0.0904	0.0144
d	(((((radi,siph),digi),((((elong,tene),cong),(rubr,ruba)),rubes)),(((uni,sac),dehi),((bullo,falco),quin))),pela);	30.385	< 0.001	< 0.001	0.0017	< 0.001

Fig. S3. Statistical testing of hypotheses concerning the phylogenetic position of *P. béii*-bearing species among the spinose species. A, topology with monophyly of *P. béii*-bearing species, **B, C,** topology of paraphyly of *P. béii*-bearing species, **D,** topology of polyphyly of *P. béii*-bearing species, **E**, topology in Newick format and each parameters.ΔlnL, the difference of the log-likelihood among the tested trees, the bootstrap proportion using RELL method (Kishino et al., 1990), expected likelihood weights (ELW) (Strimmer and Rambaut, 2002), *P* values of weighted Shimodaira-Hasegawa (SH) test (Shimodaira and Hasegawa, 1999), and the Approximate Unbiased (AU) test (Shimodaira, 2002).

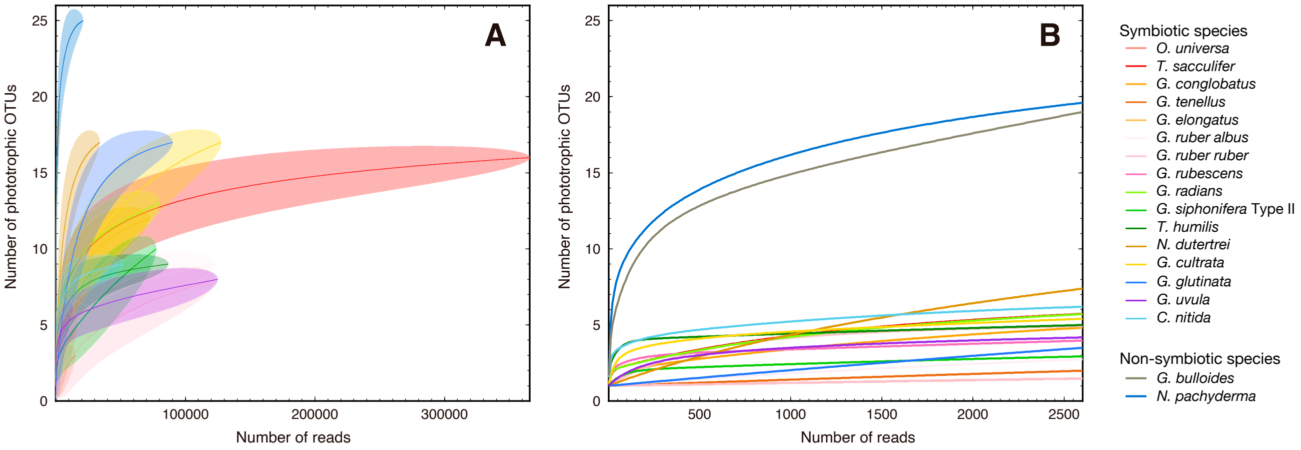

Fig. S4. Rarefaction curves of phototrophic reads for each species. Read count data are combined by species. **A,** Rarefaction curves for all phototrophic reads. Shading indicates 95% confidence interval at each number of reads. **B,** Expanded view of the rarefaction curves in **A**. The number of reads is set for the smallest sample size of a species (2602 reads, *G. bulloides*).

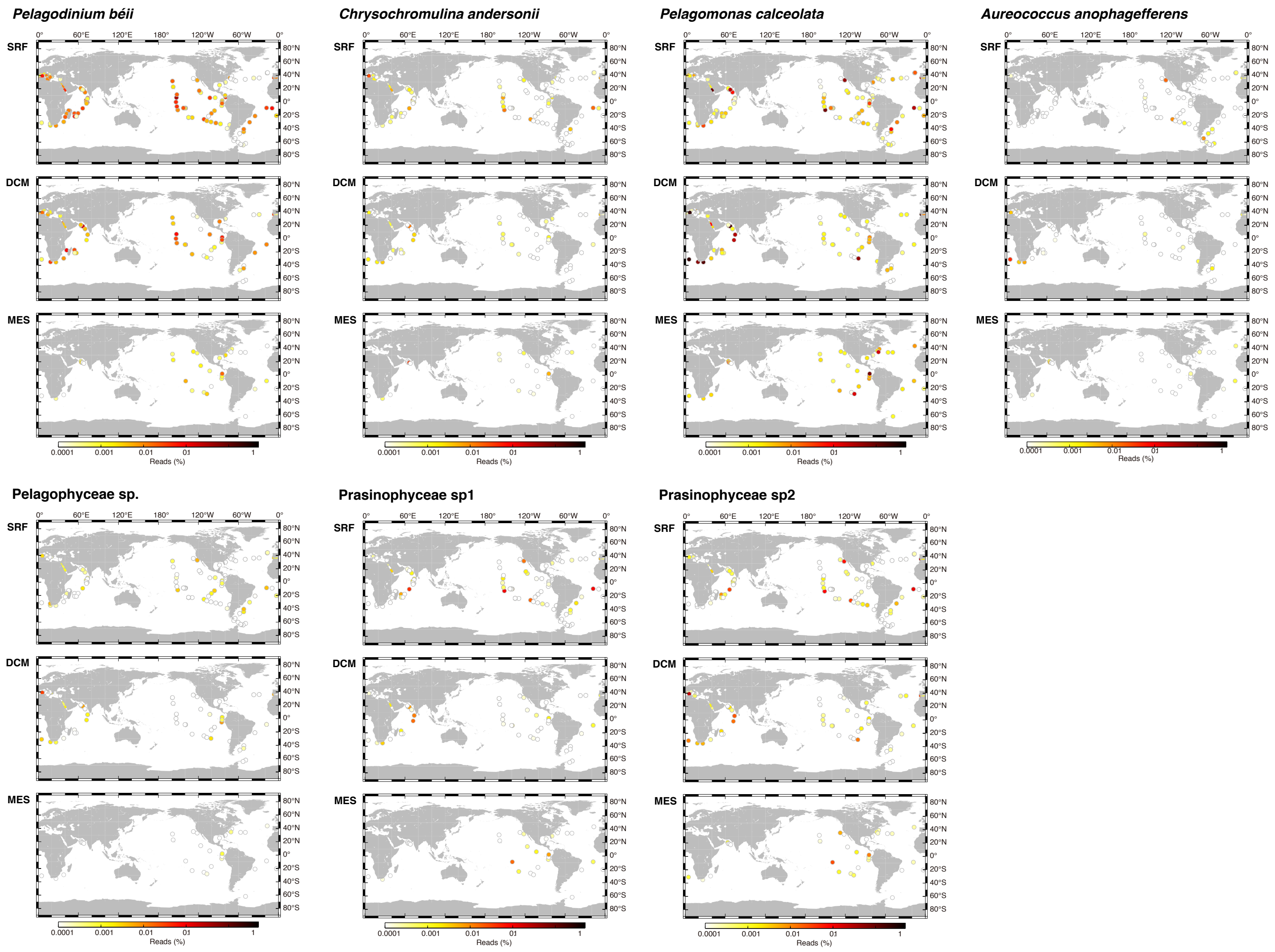

Fig. S5. Maps showing the geographical distribution and abundance of seven foraminiferal symbionts (free-living states) from the TARA Ocean database. The identical sequences obtained in this study were sorted by three depth categories; surface and mixed layer (SRF), deep chlorophyll maximum (DCM), and mesopelagic zone (MES). Proportion of the algal reads among the all eukaryotic reads at a site is represented by colors.

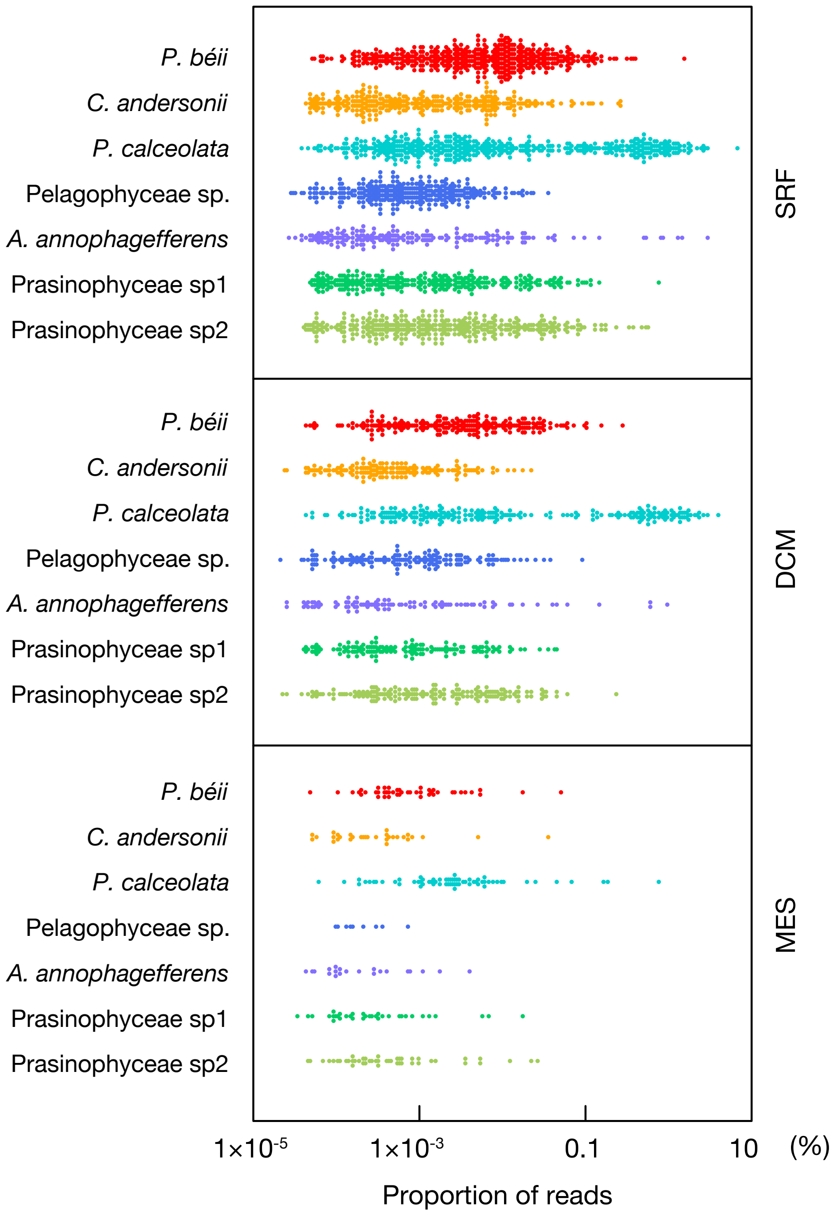

Fig. S6. Abundance plots of each foraminiferal symbiont in the TARA Ocean database by three depth categories. Surface and mixed layer (SRF), deep chlorophyll maximum (DCM), and mesopelagic zone (MES). Each plot corresponds to a sampling site of the TARA expedition shown in the map of Fig. S5.

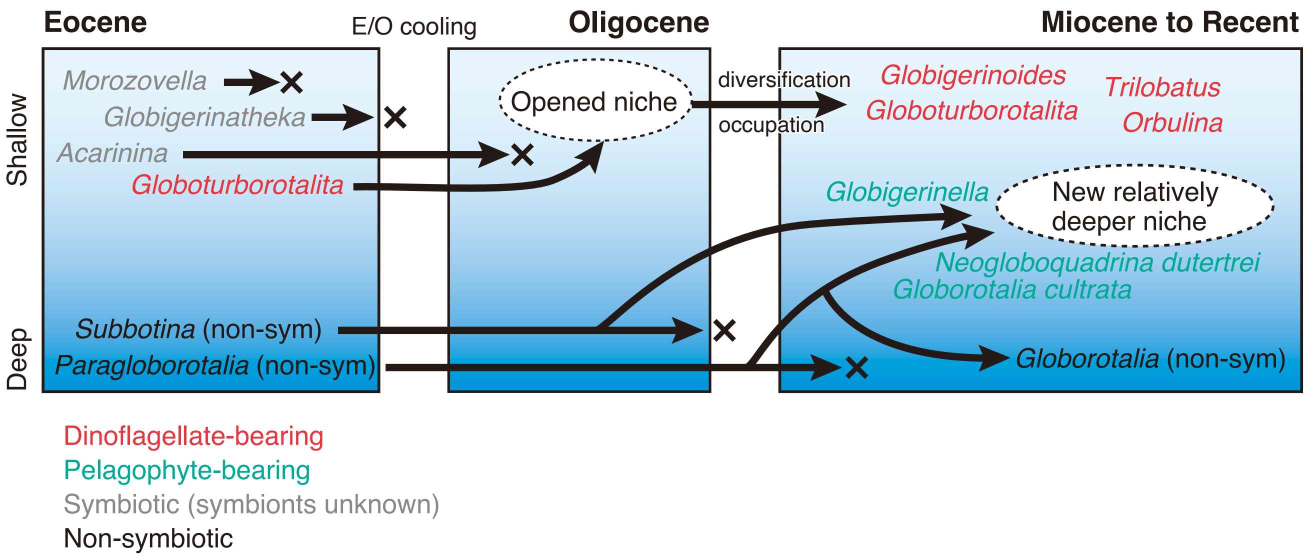

Fig. S7. Schematic illustration of planktonic foraminiferal habitats and photosymbiotic partnership through time. Surface water was occupied by older photosymbiotic lineages like *Morozovella*, *Acarinina*, and *Globigerinatheka* in the Eocene. *Globoturborotalita*, now having dinoflagellate symbiont, diversified and occupied the surface niche after the demise of the Eocene photosymbiotic lineages. Eocene muricate planktonic foraminifera (*Morozovella* and *Acarinina*) have been proposed to possess mat-forming algae as their symbionts (Gaskell and Hull, 2019). Pelagophyte-bearing photosymbiosis was repeatedly acquired after the late Miocene cooling, which could extend photosymbiotic niche vertically and horizontally.

References in supplementary materials

Gaskell, D. E. & Hull, P. M. Symbiont arrangement and metabolism can explain high δ13C in Eocene planktonic foraminifera. Geology 47, 1156–1160 (2019).

Kishino H, Miyata T, Hasegawa M. Maximum likelihood inference of protein phylogeny and the origin of chloroplasts. J Mol Evol 1990;31:151–60.

Locarnini, R. A. et al. World ocean atlas 2013. Volume 1, Temperature. (2013) doi:10.7289/V55X26VD.

Shimodaira H. An Approximately Unbiased Test of Phylogenetic Tree Selection. Goldman N (ed.). Syst Biol 2002;51:492–508.

Shimodaira H, Hasegawa M. Multiple Comparisons of Log-Likelihoods with Applications to Phylogenetic Inference. Mol Biol Evol 1999;16:1114–6.

Strimmer K, Rambaut A. Inferring confidence sets of possibly misspecified gene trees. Proc R Soc Lond B Biol Sci 2002;269:137–42.
